# Potential Therapeutic Role of HDAC Inhibitors in FUS-ALS

**DOI:** 10.3389/fnmol.2021.686995

**Published:** 2021-08-09

**Authors:** Clara Tejido, Donya Pakravan, Ludo Van Den Bosch

**Affiliations:** ^1^Vlaams Instituut voor Biotechnologie (VIB), Center for Brain and Disease Research, Laboratory of Neurobiology, Leuven, Belgium; ^2^Department of Neurosciences, Experimental Neurology, Leuven Brain Institute, Katholieke Universiteit Leuven (KU Leuven)-University of Leuven, Leuven, Belgium

**Keywords:** ALS, FUS, HDAC inhibitors, acetylation, mislocalization, LLPS, RNA, RRM

## Abstract

Mutations in the *FUS* gene cause amyotrophic lateral sclerosis (ALS-FUS). However, the exact pathogenic mechanism of mutant fused in sarcoma (FUS) protein is not completely understood. FUS is an RNA binding protein (RBP) localized predominantly in the nucleus, but ALS-linked FUS mutations can affect its nuclear localization signal impairing its import into the nucleus. This mislocalization to the cytoplasm facilitates FUS aggregation in cytoplasmic inclusions. Therapies targeting post translational modifications are rising as new treatments for ALS, in particular acetylation which could have a role in the dynamics of RBPs. Research using histone deacetylase (HDAC) inhibitors in FUS-ALS models showed that HDACs can influence cytoplasmic FUS localization. Inhibition of HDACs could promote acetylation of the FUS RNA binding domain (RRM) and altering its RNA interactions resulting in FUS maintenance in the nucleus. In addition, acetylation of FUS RRMs might also favor or disfavor its incorporation into pathological inclusions. In this review, we summarize and discuss the evidence for the potential role of HDACs in the context of FUS-ALS and we propose a new hypothesis based on this overview.

## Introduction

Amyotrophic lateral sclerosis (ALS) is the most common adult-onset motor neuron disease (Renton et al., 2014). It is characterized by the selective degeneration of motor neurons in the motor cortex, brainstem and spinal cord. This leads to progressive paralysis and the death of the patient on average 2–5 years after the detection of the first symptoms (Van Damme et al., 2017). There is currently no effective treatment for ALS. About 90% of ALS patients have no family members suffering from the same disease and are classified as sporadic ALS (sALS). The remaining 10% are considered as familial ALS (fALS) (Kiernan et al., 2011) and four causative genes explain the majority of these fALS cases (Hardiman et al., 2017). Mutations in the FUS gene account for ~4% of fALS cases (Vance et al., 2009; Renton et al., 2014). FUS pathology appears when there is no TAR DNA-binding protein 43 kDa (TDP-43) pathology (Kwiatkowski et al., 2009; Ticozzi et al., 2009) a phenomenon not yet understood. TDP-43 is the most common pathological protein as it accounts for almost 97% of ALS cases (Neumann et al., 2006; Ling et al., 2013). FUS inclusions are found in ~1% of ALS patients as well as in 9% of FTD patients (Arai et al., 2006; Neumann et al., 2006; Ling et al., 2013).

TDP-43 and FUS are both RNA binding proteins which normally reside in the nucleus, although they often shuttle between the nucleus and the cytoplasm (Zinszner et al., 1997; Tziortzouda et al., 2021). ALS-linked FUS and TARDBP mutations lead to a shift in this equilibrium resulting in the mislocalization of FUS and TDP-43 into the cytoplasm. In fact, most FUS mutations occur in the nuclear localization signal domain (NLS) (Dormann and Haass, 2013) affecting its nuclear import and subsequently resulting in cytoplasmic mislocalization of FUS (Niu et al., 2012). FUS in the cytoplasm facilitates a liquid–liquid phase separation (LLPS) process resulting in the formation of dynamic membranelles organelles, which could potentiate the formation of solid FUS aggregates (Andersson et al., 2008; Bosco et al., 2010; Lin et al., 2017).

FUS's aggregation propensity and its localization may be affected by post-translational modifications (PTM). PTMs refer to the covalent enzymatic modifications of proteins following biosynthesis which can affect their function in health and disease. Many lines of evidence suggest that PTMs of proteins linked to motor neuron diseases are powerful modifiers of their activity. For example, in TDP-43 it is well-established that acetylation can act as a strong modulator of ALS pathogenicity, affecting both TDP-43 aggregation propensity and cytoplasmic mislocalization (Cohen et al., 2015; Sanna et al., 2020). PTMs that could influence FUS-linked pathology include phosphorylation (Monahan et al., 2017), methylation (Dormann et al., 2012), ubiquitination (Farrawell et al., 2015), and acetylation (Arenas et al., 2020). In this review, we will focus on the role of FUS acetylation, modulated by histone acetylases (HAT) and histone deacetylases (HDAC) which could play a role in both FUS mislocalization and in the associated pathogenicity. We will give an overview of the data related to the role of acetylation of FUS, why it is important, and we will discuss the therapeutic potential of HDAC inhibition.

## Role of FUS in ALS

FUS is a ubiquitously expressed protein belonging to the heterogeneous nuclear protein family. Although not fully characterized, the functions of FUS are very diverse including roles in DNA damage response, in cellular stress response and in the regulation of RNA metabolism and processing (Ratti and Buratti, 2016).

**Figure 1:**
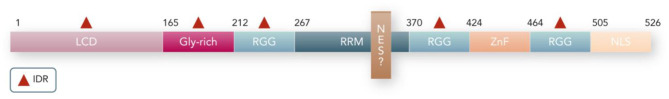
Protein domains in FUS. The FUS folded domains are: RRM, ZnF (Zinc Finger), NLS (Nuclear location signal). In addition, NES (nuclear export signal) is a putative domain that may be located in the RRM domain. The FUS intrinsically disordered regions (indicated by a red triangle) are: Low complexity domain (LCD), Glycine-rich domains, and RGGs. The number of residues that covers each domain is indicated.

FUS contains both folded domains and intrinsically disordered regions (IDRs) (Rhoads et al., 2018) (Figure 1). The folded domains consist of an RNA recognition motif (RRM) and a zinc finger. The RRM is the main known RNA binding domain, which allows FUS to play a role in many RNA related processes (Loughlin et al., 2019). FUS also has a nuclear localization signal (NLS) domain which mediates the nuclear import of FUS, allowing FUS to remain predominantly in the nucleus (Dormann et al., 2010).

IDRs consist of regions with an undefined tertiary structure, facilitating various weak molecular interactions (Kato et al., 2012). The FUS IDR domains comprises two different types: an N-terminal region rich in glutamine-glycine-serine and tyrosine residues (LCD region) (Lin et al., 2017), and multiple arginine-glycine-glycine (RGG) repeats (Ozdilek et al., 2017). The RGGs have a role in supporting RRM in RNA binding since the RRM alone is not sufficient for high affinity binding (Ozdilek et al., 2017). They are also important for toxicity and phase separation (Bogaert et al., 2018).

The roles for FUS in RNA metabolism include gene expression, splicing, mRNA transport and translation. FUS interacts with RNA polymerase II and transcription complexes, which suggests that it mediates mRNA synthesis (Masuda et al., 2016). FUS also plays an important role in RNA processing mediated by the spliceosome, thereby affecting the splicing pattern and/or abundance of about 1,000 RNAs (Lagier-Tourenne et al., 2013). Furthermore, FUS controls RNA silencing by generating small non-coding RNAs (microRNAs) (Zhang et al., 2013), and it plays a role in RNA transport (Ederle and Dormann, 2017) and translation (Yasuda et al., 2013) in the cytoplasm.

The response of FUS to general cellular stress involves FUS binding to RNA and its subsequent recruitment into cytoplasmic ribonucleoprotein (RNP) granules, such as stress granules, which are transient regulatory structures formed by RNA complexes stalled in translation (Andersson et al., 2008; Sama et al., 2014; Harley and Patani, 2020).

Last but not least, FUS can bind to DNA and participate in DNA repair by promoting single strand DNA annealing and by mediating the repair of double strand breaks (Wang et al., 2017). Indeed, FUS is recruited to DNA damage sites and plays a major role in DNA damage response pathways (Deng et al., 2014; Wang et al., 2018, 2019). Increased DNA damage was reported in cells expressing mutant FUS (Deng et al., 2014; Wang et al., 2018).

ALS-related FUS mutations leads to disturbances in several FUS functions such as RNA metabolism (Lagier-Tourenne et al., 2013; Zhang et al., 2013), DNA repair (Deng et al., 2014; Naumann et al., 2018; Wang et al., 2019), or response to stress (Dormann et al., 2010; Lenzi et al., 2015). These impairments in the normal functions of FUS are the result of two main processes associated with mutant FUS: FUS mislocalization and aberrant phase separation of FUS.

### FUS Mislocalization

FUS accumulation in cytoplasmic inclusions is characteristic of the FUS-ALS pathology and correlates with the age of onset and the severity of the clinical presentation (Dormann and Haass, 2013). Cytoplasmic accumulation can be toxic as an early study reported that motor neurons expressing FUS in which the NLS was deleted showed 3 times more apoptosis than motor neurons expressing WT FUS. Meanwhile, FUS knockouts did not show neuronal death (Scekic-Zahirovic et al., 2016). Mutant FUS exerted progressive motor deficits and cellular stress in mice through a gain of toxicity rather than a loss of function (López-Erauskin et al., 2018). This suggests that FUS mislocalization results in a toxic gain of its cytoplasmic function, ultimately leading to motor neuron death (Scekic-Zahirovic et al., 2016; López-Erauskin et al., 2018).

FUS can exit from the nucleus via an energy-dependent transport. This most probably occurs through its interaction with transporters of the exportin family using a putative nuclear export signal (NES) domain (Ederle and Dormann, 2017). However, FUS can also leave the nucleus by passive diffusion (Ederle et al., 2018). Mutations in the FUS NLS domain affect its interactions with the nuclear transport receptor Transportin-1, thus disturbing its import into the nucleus (Niu et al., 2012) and resulting in increased cytoplasmic FUS. Mislocalization also disturbs FUS self-regulatory mechanism. High nuclear concentrations of FUS will trigger alternative splicing of FUS mRNA, resulting in the removal of exon 7, a variant that can subsequently be degraded (Zhou et al., 2013). A consequence of FUS mislocalization is that self-regulation is lost. This results in the continuous production of (mutant) FUS which, in turn, will lead to further cytoplasmic accumulation (Zhou et al., 2013).

FUS binding to RNA also seems to trigger FUS mislocalization to the cytoplasm (Daigle et al., 2013). ALS *Drosophila* models with RNA binding incompetent FUS showed a predominant nuclear localization of FUS, which also did not colocalize with stress granules. In contrast, ALS-FUS that was competent to bind to RNA showed mislocalization to the cytoplasm and was recruited into stress granules (Daigle et al., 2013). In the same way, post-translational modifications impair RNA binding (e.g., acetylation in TDP43), so they can also retain such a protein in the nucleus (Sanna et al., 2020). This suggests that altering the binding of FUS to RNA could modulate FUS mislocalization and colocalization with stress granules. However, removal of the full RRM domain had no effect on FUS toxicity in *Drosophila* ALS models (Bogaert et al., 2018). Therefore, RNA binding might contribute to further FUS translocation to the cytoplasm in ALS, although further research on the exact role of the RRM in FUS mislocalization is required.

### Involvement of FUS in Liquid-Liquid Phase Separation (LLPS)

The pathogenicity of FUS in ALS could at least be partially linked to its involvement in a liquid-liquid phase separation process (Maharana et al., 2018). FUS can undergo LLPS which may further evolve into pathological aggregates in cells (Patel et al., 2015).

LLPS is a reversible process by which macromolecules separate from solvent into liquid droplets. *In vitro*, LLPS evolves through the increase in strength and order of the interactions, which could also help the conversion of a dynamic liquid droplet into a rigid solid aggregate, with a hydrogel transition phase (Boeynaems et al., 2018).

FUS is an intrinsically aggregation-prone protein (Nomura et al., 2014). However, FUS mutations, especially in the IDR residues, further promote LLPS (Patel et al., 2015). Other FUS domains also influence LLPS. For instance RGG domains enhance LCD mediated LLPS (Kang et al., 2019). However, the role of the RRM domain in LLPS is not fully understood. Some studies suggest that the RRM could be involved in FUS phase separation (Lu et al., 2017; Agrawal et al., 2019), while deleted or defective RRMs enhanced LLPS (Maharana et al., 2018; Yoshizawa et al., 2018; Mann et al., 2019), suggesting that the RRM inhibits LLPS.

The formation and dissolution of LLPS droplets in cells is tightly regulated by various cellular factors, and it is closely associated with the stress response (Saito et al., 2019). Therefore, LLPS misregulation and stress impairments may be involved in a large spectrum of neurodegenerative diseases, including ALS. In this way, post-translational modifications are rising as potential modulators in phase separation of RNA binding proteins. For instance, acetylation of TDP-43 most likely promoted LLPS through the impairment of TDP-43 binding to RNA (Cohen et al., 2015), and we know that RNA is a potential regulator of LLPS.

#### RNA in LLPS

The role of RNA is not yet completely clear (Kang et al., 2019) because some studies indicated that RNA enhanced FUS LLPS (Schwartz et al., 2013; Yang et al., 2015; Burke et al., 2016; Agrawal et al., 2019), while others showed that RNA at higher concentrations negatively influenced FUS LLPS (Maharana et al., 2018; Mann et al., 2019).

The deletion of the RRM domain seems to increase LLPS (Yoshizawa et al., 2018). This suggests that FUS/RNA interactions through the RRM domain maintain FUS in a soluble state by repressing the action of the RGG domains (Yoshizawa et al., 2018; Loughlin et al., 2019).

It is worth noting that the RRM have a low-affinity and low-specificity binding with RNA (Liu et al., 2013). In contrast, RGGs bind with a higher affinity (Iko et al., 2004; Yang et al., 2015; Ozdilek et al., 2017). This suggests that at lower RNA concentrations, RGGs would be the domains predominantly binding RNA, whereas at higher RNA concentrations the RRM would both bind RNA and inhibit LLPS. As a consequence, low RNA concentrations would induce FUS LLPS while high RNA concentrations could prevent droplet formation (Maharana et al., 2018).

Recent studies have found that RRM binds predominantly to RNA through the side chains of residues K315 and K316 (Liu et al., 2013; Loughlin et al., 2019). Indeed, mutations at these residues result in a 2-fold decrease in RNA affinity compared to the WT RRM (Loughlin et al., 2019). Furthermore, RNA-binding residues K315 and K316 appear to directly decrease RGG binding to RNA, which could reduce FUS LLPS (Loughlin et al., 2019). Therefore, the FUS RRM may bind strongly to high concentrations of RNA through residues K315 and K316, and thereby inhibit the effect of RGG on FUS droplet formation (Loughlin et al., 2019).

#### Stress Granules in LLPS

Mutant FUS showed an enhanced propensity to be recruited to stress granules compared to WT FUS (Dormann et al., 2010; Gal et al., 2011; Ito et al., 2011; Daigle et al., 2013) and mutant FUS was able to sequester WT FUS into stress granules (Vance et al., 2013). In addition, mutant FUS-containing stress granules were shown to dissolve in a slower manner after the stressor stops than WT FUS-containing stress granules (Lenzi et al., 2015). Therefore, mutant FUS impairs the cellular stress response potentially leading to pathological stress.

This pathological stress response reveals a close association of stress granules with FUS LLPS, as can be concluded from the stress granule markers present in pathological FUS aggregates (Dormann et al., 2010; Gal et al., 2011). Indeed, FUS can give rise to these stress granules through the liquid-liquid phase separation process (Hyman et al., 2014; Taylor et al., 2016). In addition, RNA and the abnormal stress response are factors that can work together to promote FUS aggregation (Burke et al., 2016). FUS binding to RNA promoted its translocation to the cytoplasm and recruitment into stress granules (Daigle et al., 2013). However, RNA binding to the RRM domain could inhibit LLPS (Yoshizawa et al., 2018; Loughlin et al., 2019), thus may result in a reduction of stress granules as well (Taylor et al., 2016). Therefore, binding of FUS to RNA promotes colocalization with stress granules but could also have a protective effect against stress by reducing liquid phase separation.

### Connection of FUS Mislocalization to FUS LLPS

Although the nuclear concentration of FUS proved to be high enough for droplets formation, only 1% of the nuclear FUS forms aggregates in living cells (Fox et al., 2017). High concentrations of RNA in the nucleus is the main factor preventing FUS droplets in this compartment. It is estimated that the nuclear RNA concentration is 10 times higher than that required to dissolve FUS droplets, thus strongly preventing FUS from undergoing LLPS (Maharana et al., 2018). In contrast, the concentration of RNA in the cytoplasm is lower, which facilitates droplet formation. When additional RNA was added to these cytoplasmic droplets, they dissolved (Maharana et al., 2018). However, it is worth mentioning that increased RNA binding has also been reported to lead to increased formation of FUS inclusions in the cytoplasm (Yang et al., 2015). Therefore, further studies are needed to determine the role of RNA in the formation of FUS cytoplasmic inclusions.

Moreover, FUS mislocalization by increasing the export to the cytoplasm or decreasing the import into the nucleus was related to higher FUS toxicity (Hofweber et al., 2018; Steyaert et al., 2018). Transportin-1 could suppress FUS droplets, and also avoid the association of FUS to stress granules (Hofweber et al., 2018). Thus, reduced interaction with nuclear importer proteins seems to increase FUS toxicity. Moreover, downregulation of proteins potentially involved in the nuclear export of FUS, such as exportin, decreased FUS recruitment into cytoplasmic stress granules, hence ameliorating FUS toxicity (Steyaert et al., 2018). Therefore, export of FUS to the cytoplasm might be linked to increased colocalization with stress granules.

In conclusion, FUS mislocalization can ultimately lead to cytoplasmic aggregation which causes disturbances in several metabolic processes and in cellular stress. Cytoplasmic FUS can undergo LLPS as the RNA concentration is lower than in the nucleus. Consequently, stress granules or other membraneless LLPS granules could be the stepping stone for the formation of pathological aggregates.

## Histone Acetylases (HATS) and Histone Deacetylases (HDACS): Potential New Targets for Therapy in FUS-ALS

The HAT/HDAC homeostasis could be altered in ALS, leading to hypoacetylation or hyperacetylation. Postmortem analysis of brains and spinal cords of ALS patients showed an increase of HDAC2 mRNA (Janssen et al., 2010). Moreover, a significant decrease in histone acetylation in FUS-ALS models suggests a higher proportion of nuclear HDACs in comparison to HATs (Kuta et al., 2020). An imbalance of the HAT/HDAC ratio usually leads to defects in DNA repair (Robert et al., 2011) and stress response (Kuta et al., 2020). In particular, mutant FUS motor neurons have defects in chromatin remodeling (Tibshirani et al., 2017), an altered stress response (Kuta et al., 2020) and reduced levels of histone acetylation (Rossaert et al., 2019). Moreover, HDAC inhibitors exerted neuroprotective effects or resulted in an improved survival of ALS motor neurons expressing mutant FUS (Guo et al., 2017; Kuta et al., 2020).

**Figure 2:**
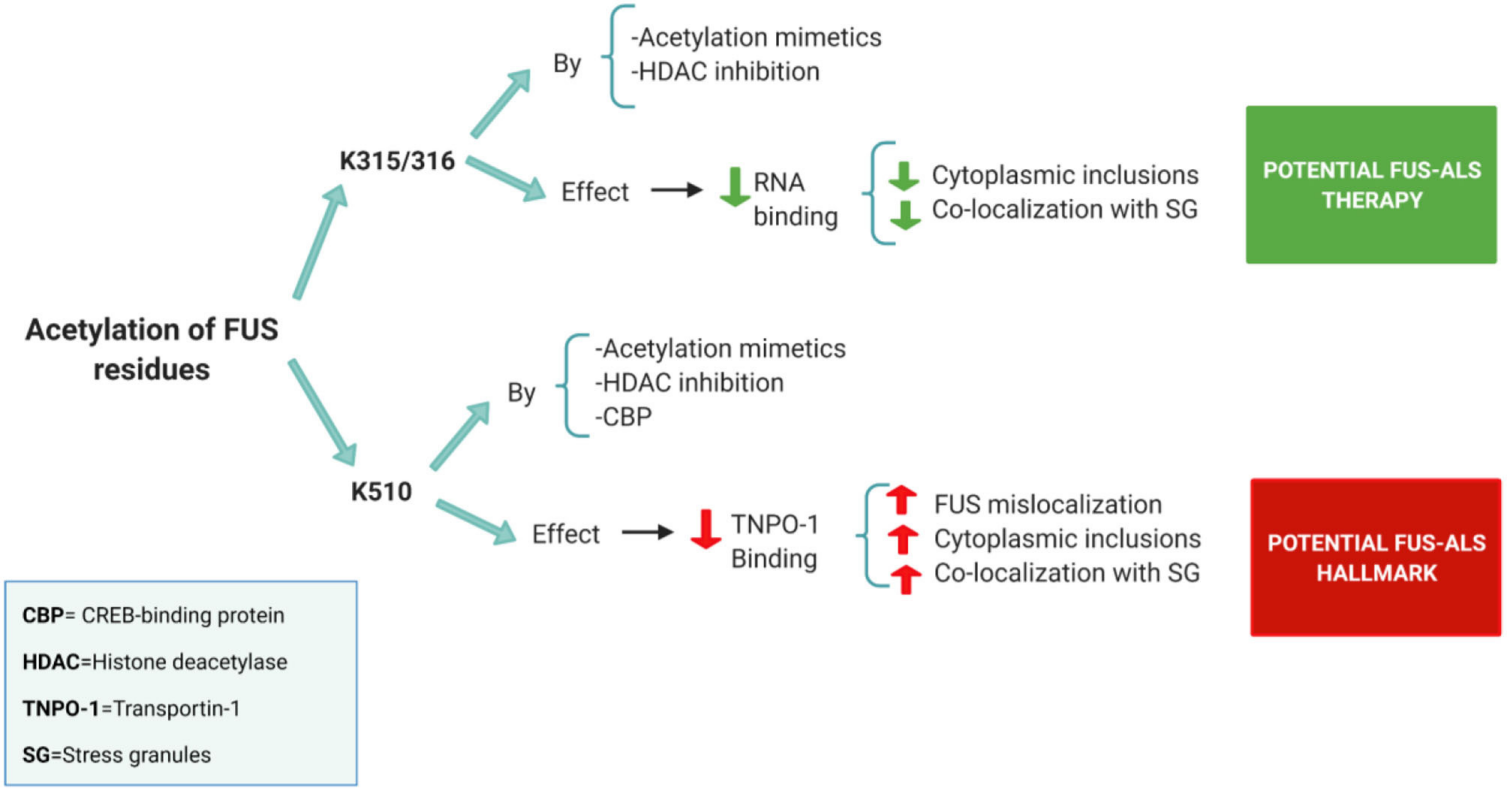
Summary of the acetylation sites in FUS and its potential effects on FUS-ALS pathology [based on the results obtained in the study of Arenas et al. (2020)]. Acetylation of the FUS RRM (K315/316) by both acetylation mimetics and HDAC pan-inhibition (using a DACi cocktail) decreased FUS ability to bind to RNA. Prevention of RNA binding reduces cytoplasmic inclusions and FUS colocalization with stress granules. Thus, FUS acetylation on RRM sites has a positive effect on FUS-ALS pathology. On the other hand, FUS NLS region (K510 site) proved to be acetylated by acetylation mimetics, CBP or pan-HDAC inhibition. Acetylation of the K510 residue results in the loss of affinity with Transportin-1 leading to increased FUS mislocalization. Thus, FUS acetylation on the NLS site has a negative effect on FUS-ALS pathology.

However, the exact relationship between HDACs and FUS is not yet clear. Historically the main function of HATs and HDACs is to modulate epigenetic on/off chromatin states controlling transcription. However, they can also reversibly acetylate/deacetylate other proteins as a direct post-translational modification (Glozak et al., 2005). In this way, three potential acetylation sites have been discovered in FUS: two of them located in the RRM domain (K315/K316) and another one in the NLS region (K510) (Arenas et al., 2020) (Figure 2).

Acetylation of the NLS region is believed to play a role in FUS mislocalization, as it has been shown to reduce the FUS interaction with Transportin-1 (Arenas et al., 2020). FUS mutants with acetylation mimetics in the K510 residue showed a significant increase in FUS mislocalization, inclusions formation and colocalization with stress granules (Arenas et al., 2020) (Figure 2). In addition, in this study, FUS displayed high levels of K510 acetylation in postmortem ALS tissues. The histone acetylase CBP/p300 appears as a major acetyltransferase at the K510 site of FUS (Arenas et al., 2020). Therefore, FUS acetylation of the NLS region might be a hallmark of FUS-ALS, and CBP could play a role further increasing this pathology (Arenas et al., 2020) (Figure 2).

On the other hand, acetylation in the RRM domain, specifically in the K315/ K316 lysine's seems to play a potential positive role in FUS-ALS pathology (Arenas et al., 2020). The RRM domain interacts weakly with RNA, but, certain residues such as K312/K315/K316 appear to form an extra-long positively charged KK loop that plays a critical role in RNA binding (Liu et al., 2013). In this way, acetylation mimetics in K315/K316 residues effectively block RNA binding resulting in a reduction of cytoplasmic inclusions and stress granules (Arenas et al., 2020) (Figure 2). Similar results have also been obtained using HDAC inhibitors (Arenas et al., 2020) (Figure 2).

Thus, acetylation appears to have a modulatory effect in the FUS-ALS pathology. However, it seems important that acetylation must be targeted to certain residues in the RRM domain to ameliorate this disease. Therefore, we will focus our review on the potential effects of FUS RRM acetylation on cytoplasmic shuttling and LLPS.

## Potential Therapeutic Role of HDAC Inhibitors in FUS-ALS

**Figure 3:**
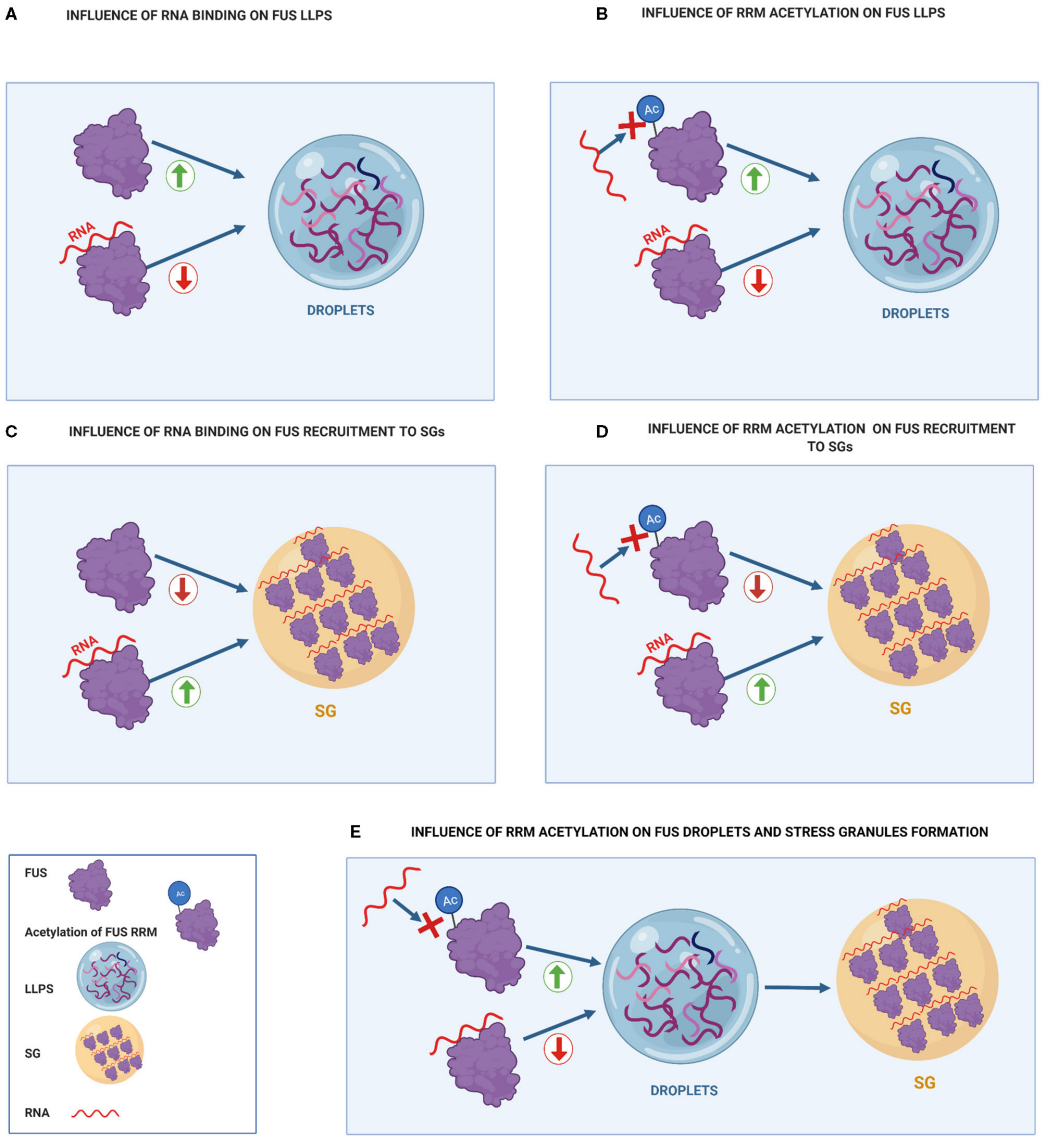
Influence of RNA binding on FUS toxicity compared to the effect of FUS acetylation of RRM residues on FUS toxicity. **(A)** Influence of RNA binding on FUS LLPS. Binding of FUS to high RNA concentrations prevents it from undergoing LLPS. **(B)** Influence of RRM acetylation on FUS LLPS. RRM acetylation prevents RNA-RRM interactions, increasing FUS LLPS. **(C)** Influence of RNA binding on FUS recruitment to SGs. RNA binding increases FUS recruitment to SG. **(D)** Influence of RRM acetylation on FUS recruitment to SGs. Acetylation of RRM domains prevents FUS from binding to RNA, decreasing FUS recruitment to SGs. **(E)** Influence of RRM acetylation on FUS droplets and stress granules formation. RRM acetylation promotes droplet formation that can further turn into stress granules.

**Figure 4:**
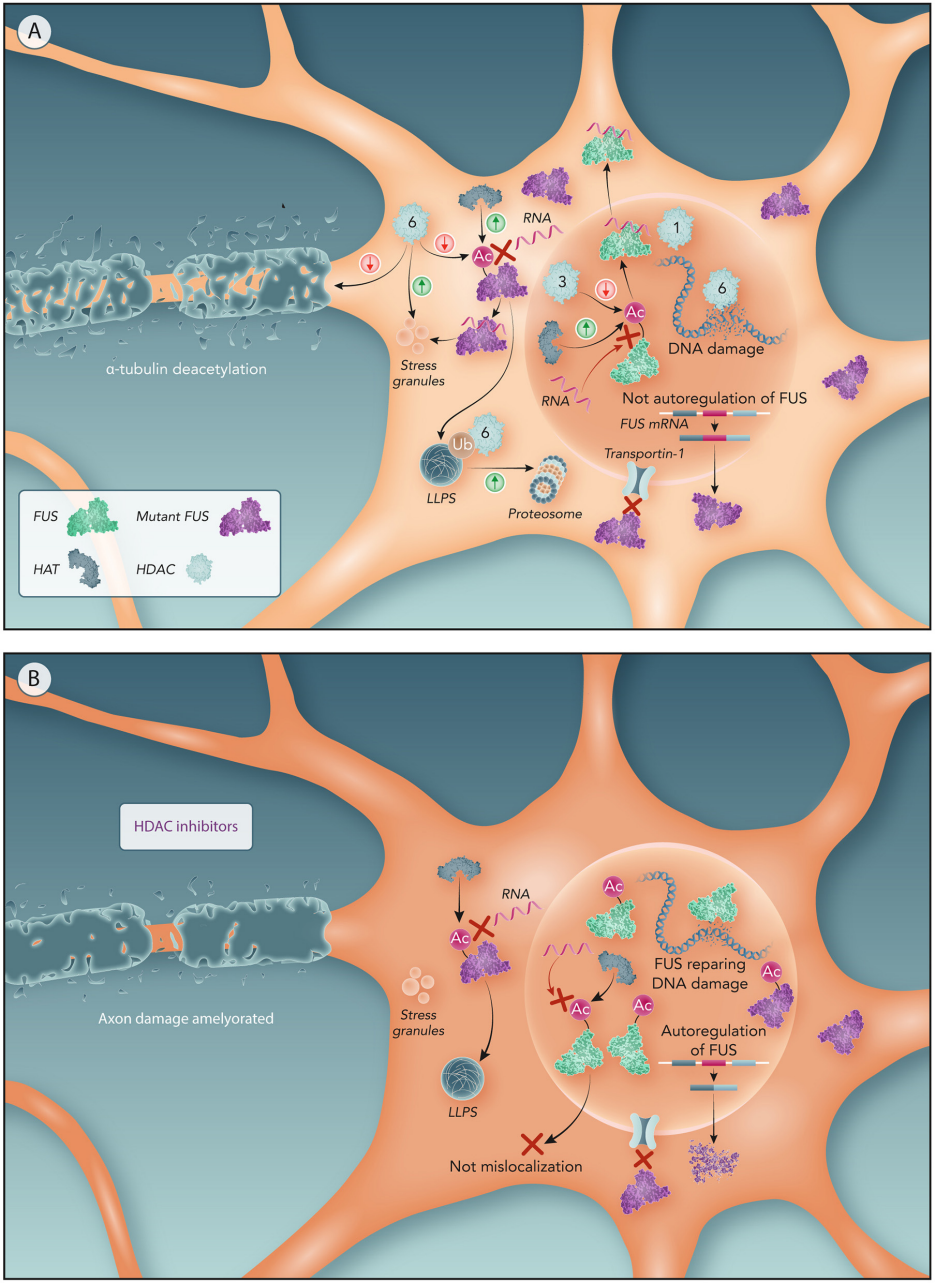
Potential molecular mechanism of HDACs to drive FUS mislocalization and related ALS features. **(A)** Motor neurons with NLS-mutated FUS. Mutations in NLS domain impairs FUS binding to Transportin-1 resulting in FUS cytoplasm sequestration. Lower concentrations of nuclear FUS not activates FUS autoregulation resulting in an overproduction of mutant FUS producing further increase on FUS cytoplasmic levels. Nuclear HATs acetylate FUS RRM preventing its binding to RNA and hence FUS egress from the nucleus. Nuclear HDACs (like HDAC3) increases FUS mislocalization by deacetylating RRM. A higher ratio of nuclear HDACs/HATs has been associated with ALS, hence driving FUS mislocalization through HDACs. Moreover, the reduction of HDAC1-FUS interactions caused by FUS mislocalization impairs DNA repair resulting in DNA damage. In the cytoplasm, HATs acetylation of the RRM can promote LLPS of FUS. HDAC6 participates in the proteasomal clearance of FUS aggregates. Abnormal cytoplasmic expression of HDAC6 impairs axonal transport and, also, generates DNA damage. **(B)** Motor neurons with NLS-mutated FUS treated by HDAC inhibitors. In absence of nuclear HDACs, nuclear FUS is only subject of the HATs effect, thereby acetylation of FUS RRM keeps FUS within the nucleus. Therefore, maintaining FUS in the nucleus reactivates the autoregulatory mechanism of FUS reducing the production of mutant FUS. Prevention of FUS mislocalization also avoids FUS recruitment into stress granules and stress impairments associated to ALS. Moreover, HDAC6 inhibition ameliorates axonal damage from α-tubulin deacetylation and DNA damage caused by HDAC6.

Post-translational modifications strategies for ALS are hypothesized to be a potential therapeutic targets (Pakravan et al., 2020). Acetylation of the RRM domain could be a valid therapeutic strategy (Figure 2). In fact, some recent studies demonstrated the effect of different HDAC inhibitors on FUS-ALS pathology (Arenas et al., 2020; Kuta et al., 2020). There are four classes of HDACs. The main nuclear HDACs belong to Class I (HDAC 1, 2, 3, and 8), and the cytoplasmic ones to Class IIb (HDAC 6 and 10) (Yang et al., 2017). Based on the results obtained using HDAC inhibition in FUS-ALS (Arenas et al., 2020; Kuta et al., 2020), we propose a molecular mechanism that could link the different HDACs to FUS mislocalization and FUS-derived toxicity (Figures 3, 4).

### Effects of Nuclear HDAC Inhibition on FUS Mislocalization and Toxicity

Both pan-HDAC inhibitors as well as HDAC1 and 3 inhibitors preserved the nuclear localization of FUS in ALS linked to mutant FUS (Kuta et al., 2020). Deacetylation by HDACs would thus contribute to the nuclear export of FUS and inhibition of nuclear HDACs was suggested to counteract the egress of nuclear FUS. Furthermore, the maintenance of nuclear FUS levels through HDAC inhibition would facilitate the FUS self-regulatory mechanism explained before (Zhou et al., 2013).

The underlying molecular mechanism of HDAC-FUS interaction was proposed to be related to the RNA binding ability of FUS. RNA-binding-incompetent FUS strongly localized in the nucleus in FUS-ALS models (Daigle et al., 2013; Liu et al., 2013). This suggests that cytoplasmic mislocalization of mutant FUS might be mediated by its RNA binding ability, proposing that FUS forms a FUS–RNA complex before being transported to the cytoplasm. Point mutations in the FUS RRM (K315/K316 residues) effectively decrease FUS binding to RNA (Liu et al., 2013), so we propose that acetylation of these residues could reduce FUS binding to RNA and thereby FUS mislocalization. Theoretically, lysine acetylation of the FUS RRM domain would inhibit the positive charge of lysine residues preventing this domain to interact with RNA, turning FUS into an RNA binding-incompetent complex that will remain in the nucleus. Further evidence on the role of acetylation on RNA binding proteins is provided by acetylation of the RRM of TDP-43 which showed to be retained in the nucleus (Cohen et al., 2015; Sanna et al., 2020). Therefore, nuclear HATs could acetylate the RRM domain of FUS helping to preserve FUS in the nucleus and nuclear HDACs could reverse this acetylation contributing to the mislocalization of FUS (Figure 4).

CBP and p300 are predominantly nuclear HATs and thus could be considered for use to promote FUS acetylation in the RRM domain and hence reduce FUS mislocalization. However, CBP was shown to acetylate significantly more the residue K510 than the rest of the FUS protein (Arenas et al., 2020). As previously mentioned, acetylation of K510 prevents FUS from being imported into the nucleus and thus would increase mislocalization in FUS-ALS (Arenas et al., 2020). Therefore, we believe that using nuclear HDAC inhibitors is a more effective therapeutic strategy than using HATs.

However, not all individual HDACs might equally contribute to this FUS mislocalization. The expression and localization of HDAC3 in the nucleus is associated to neurodegeneration (Qu and Mello, 2018). This suggests that HDAC3 could eventually deacetylate the RRM domain contributing to export of FUS to the cytoplasm (Figure 4). In fact, HDAC3-specific inhibitors (RGFP109 and RGFP966) preserved the FUS nuclear localization in FUS-ALS models (Kuta et al., 2020), suggesting that FUS mislocalization is directly associated to deacetylation induced by HDAC3. Combined inhibition of HDAC1 and HDAC3 was required to enhance nuclear localization of FUS (Kuta et al., 2020). Moreover, the neurotoxicity was higher when both HDAC1 are HDAC3 were co-expressed. As a consequence, they might also be both involved in regulating FUS (mis)localization (Bardai et al., 2012).

Regarding FUS toxicity, an inhibitory cocktail containing pan-HDAC inhibitors, such as sodium butyrate, and nuclear HDAC inhibitors like Trichostatin A, showed a significant reduction of cytoplasmic FUS inclusions in cells expressing mutant FUS (Arenas et al., 2020). Therefore, it is possible that acetylation of the RRM of nuclear FUS prevents FUS mislocalization resulting in decreased cytoplasmic inclusions and reduced recruitment of FUS to stress granules. Moreover, in TDP-43, both HDAC 1 genomic inactivation and pan-HDAC inhibitors exerted a protective role against TDP-43 ALS toxicity (Sanna et al., 2020). As a consequence, nuclear HDAC inhibitors could be a therapeutic strategy to reduce FUS toxicity in ALS.

On the other hand, given the role of FUS in DNA repair, it is expected that mutations in the NLS of FUS will lead to deficiencies in this process, as supported by the increased markers of double strand breaks in postmortem ALS tissues (Wang et al., 2017). According to the role suggested of HDACs in FUS mislocalization, nuclear HDAC inhibition is expected to cause nuclear retention of FUS promoting DNA repair. In fact, treatment with pan-HDAC inhibitor showed higher FUS recruitment to the DNA damage sites, and, hence, ameliorated DNA damage (Kuta et al., 2020). In particular, HDAC1 seems to be the main HDAC related to DNA repair by FUS. FUS was involved in recruiting HDAC1 to sites of induced DNA damage where it interacted with enzymes related to single strand break repair like AP-endonuclease (Bhakat et al., 2003). As a consequence, decreased FUS-HDAC1 interactions driven by FUS NLS mutations could result in an impairment of a proper DNA damage response (Naumann et al., 2018) (Figure 4). However, HDAC1 inhibition alone did not enhance DNA repair, unless it was combined with HDAC3 inhibition (Kuta et al., 2020).

### Effects of Cytoplasmic HDAC Inhibition on FUS Toxicity

FUS mutations caused cytoplasmic aggregates, showed toxicity related to impairments of the stress response (Aulas and Vande Velde, 2015) and disturbances in axonal transport (Yasuda et al., 2017; Guo and Van Den Bosch, 2018). In this scenario, cytoplasmic HAT/HDACs could also play a role in FUS-related toxicity.

Acetylation of the FUS RRM might enhance LLPS as was shown for the RRM acetylation of other RBPs, such as TDP-43 (Cohen et al., 2015). As mentioned before, the binding of high concentrations of RNA to the RRM inhibited FUS LLPS (Yoshizawa et al., 2018) (Figure 3A). Both FUS and TDP-43 RRM acetylation inhibited RNA binding, hence disrupting the inhibitory function of RRMs on LLPS (Cohen et al., 2015) (Figure 3B). Thus, RRM acetylation through HDAC inhibition might promote FUS LLPS, which can also favor FUS transition into membrane-less organelles, such as stress granules (Taylor et al., 2016) (Figure 3E).

On the other hand, acetylation of the FUS RRM could reduce the colocalization of FUS with stress granules (Figure 3D). This may happen because RNA binding to RRM promotes FUS recruitment into stress granules (Figure 3C) (Daigle et al., 2013). In this regard, FUS mutants with acetylation at K315/K316/K510 residues show significantly fewer cytoplasmic inclusions and less colocalization with stress granules than FUS mutants with acetylation at K510 residue (Arenas et al., 2020). Therefore, when FUS is mislocalized, it appears that acetylation of the RRM (K315/K316) has a beneficial effect in both FUS inclusion formation and association with stress granules. Thus, cytoplasmic HDACs inhibitors are suggested as a possible therapeutic option to mediate FUS toxicity.

HDAC6 is mainly a cytoplasmic enzyme and its inhibition did not preserve FUS in the nucleus (Kuta et al., 2020). However, it appears that HDAC6 inhibition could have important neuroprotective effects (d'Ydewalle et al., 2012). HDAC6 could play an important role in facilitating proteasome clearance of aggresomes containing misfolded proteins by binding to ubiquitinated residues (Boyault et al., 2007). Indeed, HDAC6 has been shown to interact with TDP-43 under both normal and pathological conditions (Cohen et al., 2015). However, in TDP-43 ALS mutants, the interaction with HDAC6 seems to be stronger, as western blot have shown a band indicating colocalization of HDAC6 with mutant TDP-43 but not WT one (Hebron et al., 2013). Moreover, silencing of HDAC6 using siRNAs significantly increased the aggregation of TDP-43 (Cohen et al., 2015). In addition, knockdown of HDAC6 enhanced protein aggregation of Superoxide Dismutase 1 (SOD1) and this prolongates the survival of the SOD1 mice (Gal et al., 2013). Thus, HDAC6 might also promote clearance of FUS aggregates, and as such, HDAC6 inhibition would result in increased FUS aggregates (Figure 4).

Furthermore, HDAC6 is a key component of stress granules and plays a crucial role in stress granules formation (Kwon et al., 2007; Saito et al., 2019). It was shown that pharmacological inhibition of HDAC6 abolished stress granule formation in mouse embryo fibroblasts (Kwon et al., 2007). HDAC6 could promote FUS colocalization to stress granules through deacetylation of the FUS RRM domain (Figure 4). Therefore, HDAC6 inhibition is expected to prevent FUS recruitment to stress granules (Figure 4).

In addition, HDAC6 also influenced other FUS-related toxicities associated to ALS. HDAC6 inhibition using Tubastatin A restored axonal transport deficits in motor neurons derived from both FUS-ALS (Guo et al., 2017) and TDP-43-ALS patients (Fazal et al., 2021).

Tubastatin A also ameliorated DNA damage in FUS-ALS cells. This is due to the interaction of HDAC6 with a key protein involved in DNA mismatch repair, MutL homolog 1 (Zhang et al., 2019; Kuta et al., 2020) (Figure 4).

In conclusion, HDAC6 has a protective function against proteotoxicity (Boyault et al., 2007). However, HDAC6 can generate defects in both axonal transport and DNA repair (Guo et al., 2017; Zhang et al., 2019). Therefore, HDAC6 could be a main target for the treatment of ALS toxicity caused by mislocalized (mutant) FUS.

## Conclusions

General inhibition of HDACs can effectively prevent FUS mislocalization (Kuta et al., 2020) and ameliorate FUS-ALS toxicity (Arenas et al., 2020; Kuta et al., 2020). Moreover, it was already proven that acetylation plays an important role in modulation of the location of others proteins such as Tau (Tseng et al., 2017) and TDP-43 (Sanna et al., 2020). For TDP-43, it was indeed shown that RRM acetylation mimetics can effectively retain TDP-43 in the nucleus (Sanna et al., 2020). Given the similarity between FUS and TDP-43, acetylation of the RRM of FUS is suggested to act in a similar way.

Based on previous studies on FUS acetylation and known acetylation effects on other neurodegenerative proteins, we speculate that acetylation has a double action on FUS-ALS pathology. On one hand, acetylation of the NLS region (K510 residue) can increase FUS mislocalization as it reduces FUS-Transportin interactions. In this way, FUS mutants with acetylation mimetics in the K510 residue showed higher mislocalization and an increase also in cytoplasmic inclusions and colocalization with stress granules (Arenas et al., 2020).

On the other hand, acetylation of the RRM region (K315/K316) might prevent RNA binding leading to FUS nuclear localization. Indeed, studies using HDAC inhibitors have shown an increased localization in the nucleus of FUS (Kuta et al., 2020). However, additional effects of FUS RRM acetylation should be considered since RNA deficient FUS formed cytoplasmic aggregates (Maharana et al., 2018). Moreover, the potential role of acetylation in aggregation of RNA binding proteins is also supported by a previous study showing TDP-43 aggregation upon HDAC inhibition (Chen and Cohen, 2019). Surprisingly though, HDACs inhibitors have showed to decrease both cytoplasmic inclusions and co-localization with stress granules (Arenas et al., 2020). This could be because acetylation of the FUS RRM reduces mislocalization and this also reduces the cytoplasmic toxicity of FUS. In addition, binding of FUS to RNA promotes its recruitment to stress granules, so acetylation of the RRM could be reducing FUS colocalization with stress granules.

As of today, it has not yet been proven that RRM acetylation produces both a reduction in FUS mislocalization and its associated cytoplasmic toxicity. Acetylation mimetics in K315/K316 did not prevent mislocalization but reduced FUS cytoplasmic inclusions and colocalization with stress granules (Arenas et al., 2020). However, it has been proven that RRM acetylation effectively reduces TDP-43 mislocalization and cytoplasmic inclusions formation (Sanna et al., 2020). Therefore, recent studies provide evidence for the beneficial effects of FUS RRM acetylation (K315/K316 residues).

It is worth pointing out that HDAC inhibitors have been shown to acetylate not just the K315/K316 but also the K510 residue (Arenas et al., 2020), though the use of HDAC inhibitors has thus far shown solely beneficial effects in FUS-ALS. An alternative option would be to promote K315/K316 acetylation through the use of histone acetylases. However, CBP/p300 has been shown to have a major acetylation site at K510 (Arenas et al., 2020). Therefore, it seems that presently the optimal therapy would be to use HDAC inhibitors. However, It is also worth mentioning that HDAC inhibitors are currently being studied in ALS associated with *FUS* or *TARDBP-43* mutations, so it is not clear whether this therapy would also be effective in sporadic ALS cases.

In conclusion, FUS acetylation through HDAC inhibitors is presented as potential therapeutic strategy for FUS-ALS (Figure 4), although the exact underlying relationship between HDACs and FUS pathology is not yet clear. Therefore, research on the individual effects of different acetylation sites on FUS mislocalization and aggregation is an interesting avenue for future research. In addition, more studies testing the effects of selective HDAC inhibitors on FUS-ALS is critical to minimize the side effects of general HDAC inhibition.

## Author Contributions

CT wrote the article. This was checked by DP and LV. All authors contributed to the article and approved the submitted version.

## Conflict of Interest

The authors declare that the research was conducted in the absence of any commercial or financial relationships that could be construed as a potential conflict of interest.

## Publisher's Note

All claims expressed in this article are solely those of the authors and do not necessarily represent those of their affiliated organizations, or those of the publisher, the editors and the reviewers. Any product that may be evaluated in this article, or claim that may be made by its manufacturer, is not guaranteed or endorsed by the publisher.
